# Biometrics and Knowledge Management Information Systems

**DOI:** 10.1007/978-981-13-2282-2_11

**Published:** 2018-09-18

**Authors:** Jeffrey Zheng, Chris Zheng

**Affiliations:** School of Software, Yunnan University, Kunming, Yunnan China; 2 Key Laboratory of Software Engineering of Yunnan, Yunnan University, Kunming, China; 3 Tahto, Sydney, Australia

**Keywords:** Biometrics, Complexity, Hierarchical organization, Feature classification, Content-based image retrieval

## Abstract

Biometrics and knowledge management information systems are two important fields in recent years to attract wider attentions from different social groups. This chapter explores the use of hierarchical construction linking with biometrics applications and knowledge management information systems. The key issues are discussed and a sample case of information acquisition in content-based image retrieval system has been illustrated.

## Introduction

Biometrics has attracted people attention in recent years due to terrorist attack and rapid scientific development and advanced information technology. In the twenty-first century, one of the most significant achievements in biology decodes a full list of gene codes of human DNA sequences. Using advanced pattern recognition technology, it is now convenient to make real-time face verification and fingerprint identification.

In general, all quantitative measures of living objects and activities from different sources including biology, anatomy, sound, photo, electronics and nerve pulse could link to biometrics. In such extremely complicated fields and areas, if we can efficiently acquire essential information to be manipulated by knowledge management information systems, then this mechanism will play an important role in the practices of applied biometrics. Useful concepts, methodologies and software/hardware toolkits in the direction will be invaluably helpful biometric applications in practical environments.

To resolve real-world problems, it is useful to apply system engineering schemes using analysis and synthesis mechanisms. In this chapter, hierarchical construction will be used as a framework to represent biometrics and knowledge management information systems. The original concepts and methodologies used in the chapter come from an established theoretical construction of dynamic systems conjugate classification and transformation [1–3]. Main algorithms and methods from the concepts have been implemented into software packages in advanced image analysis, content-based image retrieval and image understanding systems.

Using these concepts and methodologies in biometrics is a new application. The author would like to have this opportunity to sincerely discuss the possibility with other experts of the field in detail.

## Different Complexity Issues in Biometrics Applications

Different measurement may have variant forms and contents in practical biometrics applications. In a measure space, measure data set can be relevant to length, position, angles, time and other basic measurable quantitative. Using dimension number of geometric spaces representing different biometrics objects has been shown extremely useful in many applications. Very rich contents can be observed through representatives of biometrics measures.

**Infrared Detector for SARS detection (1D body temperature** $>\mathbf{38}\,^{\circ }{} \mathbf{C}$)

In protecting SARS virus distribution process, infrared detectors installed on the major channels of airports, stations and customs played active roles in indirectly measure body temperature whether higher than $38^{\circ }$. This process has significantly reduced the SARS virus fatal distributions.

**DNA sequence (1.5D sequence)**

A DNA sequence is composed of four types of gene codes forming of conjugate pair linear structure. Since the sequence itself has very complicated combination characteristics and also local grouping properties, this makes structure much more complex than simple 1D linear sequence [4].

**Face identification and early breast cancer detection (2D)**

In most image analysis systems, especially face identification and early breast cancer detection systems use of 2D features in manipulations. In larger applications or data sets, those feature spaces are very complicated.

**CT scanning and reconstruction (3D and higher D)**

Using modern CT scan medical imaging equipments, it is feasible to reconstruct 3D images from multiple 2D image slice sequences to represent complicated projection and dynamic properties of interested areas and organs. 3D visualization has much more complicated properties than 2D image visualization process.

**Retinal analysis and synthesis (higher D nerve network)**

The detailed principles of retinal nerve network in human vision is not fully understood. But their biological structures are well recognized by interconnected nerve networks. This type of connectivity is much higher than three dimensions. The corresponding symptoms of distributions among brain surfaces and visual simulations indicate hierarchical structures in optical nerve systems naturally [5].

**Abstract Thinking (Super Hypercomplex Cells)**

The capacity of abstract thinking may belong to super hierarchical organizations of nerve systems. If there are real nerve objects, this structure could be super hypercomplex cells or their superposition on extensive hierarchy [5].

From a certainty viewpoint, lower dimension cases have more certain properties than higher dimensions. In addition, higher dimension structure expressed abstract properties with more variables and richer possibilities in real-world cases.

## Proper Concepts, Methods and Useful Toolkits

Using modern mathematical toolkits, concepts and methods such as geometric topology and combinatorial topology, it is feasible to use basic analysis on neighbourhood relationship of kernel structure to partition complicated systems into non-reducible invariant characteristics base family. Using non-reducible bases as generators, it is possible to apply synthesis techniques to rebuild complicated systems in certain forms [6]. In invariant and singularity analysis relevant applications, global topologic characteristics play core roles using modern mathematics analysis toolkits [7]. Since connectivity belongs to one of the topological properties, higher dimensional geometric problems could be represented as graph problems or other forms to use common probability and statistical methods for practical calculations to resolve the equivalent problems in certain degree [8]. It does not matter how to represent a certain problem in detail, and abstract concepts could be always represented as lattice structures.

After systematic analysis of modern knowledge management information systems in concepts, principles and operational levels, a useful kernel structure Concept Cell Model for knowledge management using directed acyclic lattices in hierarchical constructions has been proposed for base construction toolkits of representation [9, 10]. The model can distinguish two similar lattices of three essential concept levels in different abstract structures as building lattice constructions:

- Time Invariant Structure: Descriptive Knowledge Lattice (Tacit, Implicit, Explicit)
- Time Variable Structure: Procedure Knowledge Lattice (Start, Operation, Finish).

Undertaken hierarchical construction, it is convenient and efficient to represent knowledge systems in information request, abstract representation, categories, organization and other statistic and dynamic application requirements.

Concept cells in hierarchies can efficiently represent from real measurement data sets to higher levels of conceptual networks to represent application systems as multiple levels of organizations. This provides an operational knowledge management framework to flexibly support from user cases, abstract design, and implementation and operation requirements for system engineering practices. By applying conceptual categories, it is feasible to construct useful application systems with powerful self-organization and self-learning capacities in wider engineering and social environments.

**Fig. 1:**
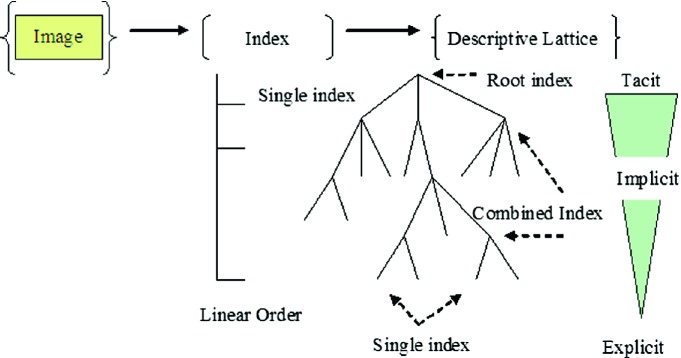
Descriptive lattice in hierarchical representation

To easily understand the main point, it is convenient to show an example to represent a partial structure in implemented content-based image retrieval systems using hierarchical concept structures shown in Fig. 1.

In the construction, a single index represents specific content-based information extracted from an image. A set of images needs to correspond to a set of indexes, respectively, and is organized as a list. It is convenient to use a multiple hierarchy to organize the list of single indexes as its end nodes. Each intermediate node can be established as a group of multiple indexes with strong similarity properties in their contents as a combined index. By this way, a root node can be established by combined individual nodes and intermediate nodes to be the representative of the whole set of indexes. Three types of information can be distinguished as follows:

- Single index: individual information explicit
- Combined index: group information implicit
- Root index: whole information tacit.

Using descriptive lattice structure in multiple levels of representations, complicated content-based image retrieval system can be mapped to a multiple layout network structure. It provides efficient organization to do information acquisition and organization linking with individuals, groups and the whole in information network construction.

While search operation, the current index will check from root (tacit node) to get the best match through combined indexes (implicit nodes) and single indexes (explicit nodes) to obtain the best-matched cases in hierarchy. Using best match information, a selected image group will be determined as output results.

**Fig. 2:**
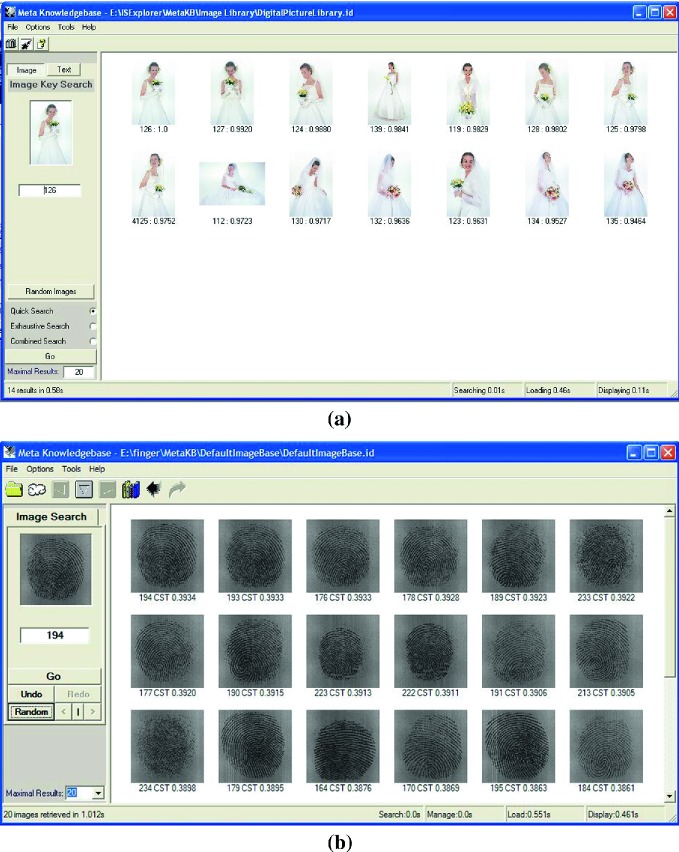
Search results: **a** Brides; **b** Fingerprints

In Fig. 2, two sets of implemented results on brides and fingerprint verification are provided to illustrate visual qualities of retrieved output results. The 125th bride image is selected and a list of similar brides as retrieved results. The 194th fingerprint image has been selected as a query example, and the output result is shown in right panel and arranged by similarity from higher to lower values in relation to the best 20 matched images from the image database in which the 194th, 193rd and 195th images are strong relative fingerprints from the same person.

**Fig. 3:**
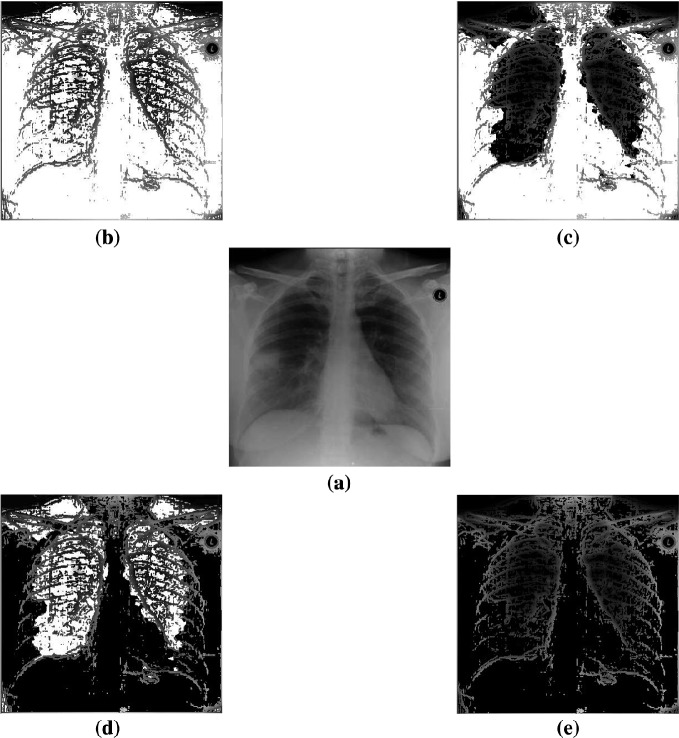
Four image enhancements on SARS image (**a**–**e**); **a** Original; **b** Positive enhanced; **c** Valley enhanced; **d** Hill enhanced; **e** Negative enhanced

**Fig. 4:**
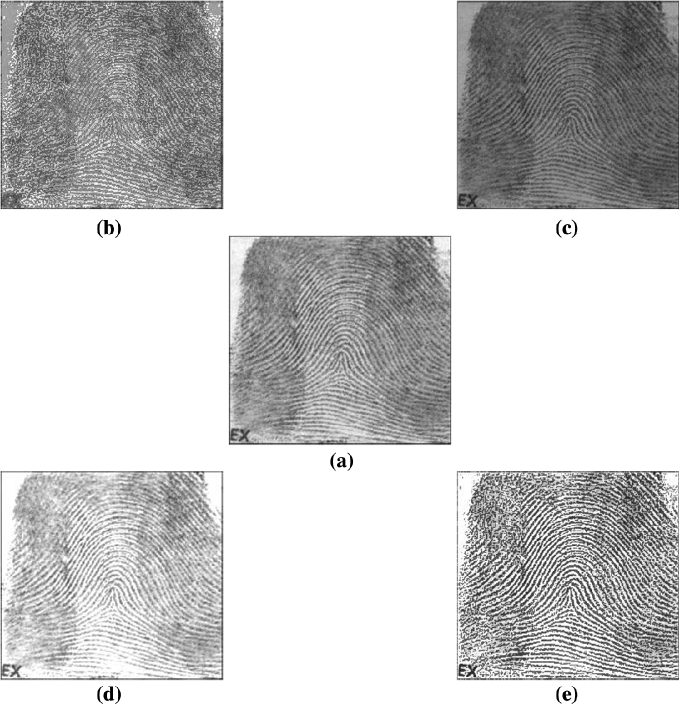
Four image enhancements on fingerprint image (**a**–**e**); **a** Original; **b** Positive enhanced; **c** Valley enhanced; **d** Hill enhanced; **e** Negative enhanced

**Fig. 5:**
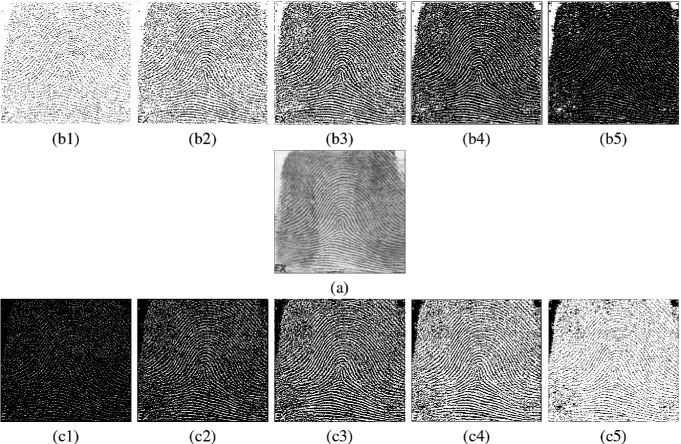
Ten enhanced results of a fingerprint image (**a**–**c**); **a** Original; **b1**–**b5** Hill enhanced; **c1**–**c5** Valley enhanced; **b1**/**c1** $\alpha$ = 30; **b2**/**c2** $\alpha$ = 80; **b3**/**c3** $\alpha$ = 128; **b4**/**c4** $\alpha$ = 160; **b5**/**c5** $\alpha$ = 220

Two sets of image processing results are shown in Figs. 3, 4 and 5. In Fig. 3, four enhanced results on an original SARS image are selected. In Figs. 4 and 5, various results of a fingerprint image are processed in different parameters under special enhanced functions.

## Demand in Future Society

From biometrics measure viewpoint, measure data itself can be very accurate and crystal certain as numeric values. However, through hierarchical construction, more uncertainty will appear as higher level contents. Complicated interconnections will be linked with simply single measures to complicated global organization. Using hierarchical construction, it is feasible to organize single, group and whole information through network construction to cover wider applications.

In rapid development of web-based network, high-speed interactive facility and quick connections have changed traditional concepts and methods significantly. It is a convenient approach to use knowledge management information system to do information acquisition, intelligent analysis, combination and synthesis.

Hierarchical operations become the most advanced parts of optimal control and best operational strategies. In the current application environment, fast, convenient and efficient design and implementation can get wider applications in many fields. It can be expected to use automatic and intelligent methodologies to complete complicated issues, especially on complex and time consumed design processes. Facing of many practical applications, simple and unified concepts can help larger dynamic system in forming stable structures. Global interactive connection and their evaluations will be helpful for social environment in high speed and sustainable development.

## Base Strategy of Development

Any theoretical scheme cannot ensure itself in practice operations successfully without carefully matching environment requirements. In current social and economic conditions, it is more important for biometrics to make a positive impact on social economy to help the existing developments. Market-oriented mechanism can be used to resolve key problems in applications. It is most important to identify core technology in the application and collect the required energies and resources to attack it resulting in significant impact.

In knowledge management information systems, content-based acquisition, representation, indexing and retrieval components are the core components for automatic organization and high-efficient retrieval. Ultra-fast and accurate retrieval technology for databases and meta-knowledge bases can be widely used in many applications to satisfy information acquisition, extraction, categories, and organization, storage and retrieval requirements. Under global web-based environment, hierarchical organization of knowledge management systems and biometrics will be further refined and developed in health environment.
